# Parenting and parent–child home practice during the COVID-19 pandemic: a case in central China

**DOI:** 10.1038/s41598-023-45726-8

**Published:** 2023-10-31

**Authors:** Jinjin Lu, Minghan Zhang, Muzi Yuan

**Affiliations:** 1 Department of Educational Studies, Xi’an Jiaotong-Liverpool University, Suzhou, 215123 China; 2 School of Psychology and Cognitive Science, East China Normal University, Shanghai, 200062 China

**Keywords:** Psychology, Health care

## Abstract

The current study aims to explore parents’ perceptions of parenting and parent–child activities at home with children aged 3–6 during the pandemic in China. A parenting survey was conducted to investigate parental role, age, educational background, work productivity, and different parenting categories during the lockdown period. We also examined the experiences of young children’s participation in extracurricular activities before and after the stay-at-home policy was implemented as well as their activities with parents during the lockdown period. The results showed that parents’ work productivity affected their perceptions of well-being during the COVID-19 pandemic. Children spent less time on extracurricular activities during the lockdown period, but some particular activities increased in frequency, especially academic extracurricular activities.

## Introduction

The Coronavirus 2019 (COVID-19) pandemic and subsequent quarantine measures have brought significant challenges to parents and their children. In the lockdown period, parenting styles, childcare demands, time spent on children, and parent–child activities might have differed from those in the past. For example, family losses and financial suffering are now associated with parents’ higher levels of stress than before the pandemic^1,2^. Fisher et al.^3^ found that parents’ job losses, income reductions, and other related mental problems directly influenced their quality of life during the pandemic. This may become a global issue for those working from home. For jobholders, the ‘Work From Home’ policy increased parents’ time spent on childcare and assisting children’s schooling, leading to more pressure, stress, burnout, anxiety, and depression^4–6^. Marginalised groups and single-parent families may have been more vulnerable during the lockdown^7^.

Several studies have shown that globally parental mental health has been severely influenced by the COVID-19 pandemic. In the United States (US), Twenge and Joiner^8^ confirmed that younger adults and children were three times more likely to experience moderate to severe mental distress than before COVID-19. Similarly, Patrick et al.^9^ found worsening parental mental health. One of the negative consequences of deteriorating parental mental health might be the low quality of childcare and nurturing at home^7^. Besides the US, increased concerns about parental stress were also found in Japan^10^, Italy^11^, and Singapore^12^. In terms of parenting^12^, revealed that parental stress was a mediator between the perceived impact of COVID-19 and parent–child relationships and between the impact of COVID-19 and harsh parenting. In China, scholars found that parents’ work productivity at home and childcare time were closely related to parent–child communication and physical health during the lockdown period^13,14^.

During quarantine, children experience stress, which, consequently, influences their well-being. Marginalised families and children would experience poverty, homelessness, poor housing quality, limited access to technology, family violence, and financial issues that might impact young people during the pandemic (Organisation for Economic Cooperation and Development^15^. Jiao et al.^16^ found that young children and adolescents experienced high levels of stress, uncertainty, fear, and distraction during quarantine. How parents and children perceived the pandemic and their home activities was also associated with their well-being^11^. In Canada, scholars found that children who spent more time with their parents and were actively engaged in home activities experienced less stress and depression^17^. Lu^13^ demonstrated that children who spent more time with siblings and parents in daily communication and physical activities were both mentally and physically healthier. These studies clearly state that the pandemic has influenced families and children’s lives, and parenting and childcare activities during the pandemic influenced both parents and their children’s mental health.

Young children’s extracurricular activities have also been influenced by the COVID-19 pandemic. Due to the lockdown policy, parents and children stayed at home, and extracurricular activities were reduced, modified, or cancelled, which affected their family lives. For example, McCormack et al.^18^ reported that Canadian children appeared to switch to more indoor physical activities, and their time spent in public spaces decreased. Children’s participation in extracurricular activities is closely linked to their parenting style^2^. Lareau^2^ claims that parents’ financial status significantly influences the development of children’s social and communicative skills, which could help them in the long term. In particular, the parenting style of working families is to support children with love, care, and food, whereas the role of extracurricular activities has not been emphasised. Compared with their Western counterparts, Chinese parents, in general, have high expectations of their children,thus, they prefer extracurricular activities that could improve children’s academic skills in their future (e.g., language, literacy, and numeracy;^19^. Therefore, the current study also investigated the changes in extracurricular activities endorsed by Chinese children before and during the lockdown period to better understand the influence of the COVID-19 pandemic on their family lives.

### Conceptual framework

This study is underpinned by Bronfenbrenner’s^20^ ecological system theory, which explains how individual children, their families, and environments interact with one another to influence their development. This theory suggests that studying the development of children in multiple environments is essential. When participating in home and social activities, children are not passive receivers; they receive only outlier reinforcement. Instead, they actively respond to the influence of the surrounding environment^21^. The ecological system theory emphasises a bidirectional influence between adults and children. In a more recent study, a systematic model was developed to explain how a ‘cascading process involves caregiver well-being and family processes (i.e. organisation, communication, and beliefs)’^22^, p. 361). This model explains how family, individuals, and social environment affect parents and children when they experience challenges and vulnerable situations^22–26^.

In this study, we adopted the systematic model and five key principles evidenced in Prime et al.^22^’s study to explore how Chinese parents and children functioned in a vulnerable and stressed situation during the pandemic. First, when the uncertainties and urgent stuff were emergent (COVID-19), children and parents were influenced by various factors, such as financial status, social distancing policies, and child-parent relationships. These negative consequences may result in emotional and behavioural problems in children. They had to stay with their parents at home because of safety issues during COVID-19, which had further negative effects. Without peer collaboration and communication, this may also influence academic studies and social interactions. Parents without enough social and financial support, together with poor interaction with their children, might experience distress and have mental health issues, affecting their well-being. All these stressors come to a family, and the whole family system (i.e., parent–child relationships, marital relationships, and family well-being) might become dysfunctional. Most importantly, this conceptual model discusses family resilience as an essential variable. Some families might experience more social disruption, which could easily lead to negative behavioural outcomes, particularly during the pandemic period. In this case, the extent to which the families suffered from the challenges they faced could differ. Those with a higher level of vulnerability find it more difficult to adapt to a new environment and, consequently, have a lower level of resilience to resist the risks faced by the family during COVID-19.

The proposed conceptual framework has been demonstrated in previous studies, which found a significant impact of COVID-19 on families in a global context. Although the pandemic has had a significant negative impact on people’s mental health and psychological changes^7^, little information has been gained on parental behaviours, particularly their involvement in parenting and home activities with children during these challenging times. In addition, most previous studies have been undertaken in a Western social context, whereas little is known about the social context of the dynamic Chinese family structure. Parenting behaviours and perceptions may differ as Chinese families have their own cultural norms, rituals, and parenting styles. Moreover, the Western media reported that the first case of COVID-19 was supposedly in Wuhan; thus, Chinese people have experienced a social distancing policy for a much longer period than those in the West. Therefore, further exploration of parental perceptions of parenting, childcare, and children’s extracurricular activities in Chinese families is required.

### The current study

Parent–child relationships, parenting roles, and home activities might be different from those in the West (e.g.^13,19,27,28^. Due to the unique Chinese sociocultural phenomenon, mothers and fathers play different roles in parenting and home activity engagement with children. That is, mothers are stricter and usually have high expectations of children’s academic performance, but fathers are more easygoing and supportive of children’s physical development^27^. Lu ^19,28^ believes that Chinese children have limited time and are less motivated to develop an extracurricular activity if it is not related to academic studies. This study focused on parenting and parent–child activities in Chinese families with preschool-aged children. Specifically, we would like to see whether parental role, age, educational background, and work productivity were related to parenting behaviours and perceptions during the lockdown period and whether children’s extracurricular activities would be altered in terms of both the time spent on the activities and the types of activities that they engaged in. Home activities and parenting experiences were examined based on parents’ self-reports to gain a better understanding of how the family system reacted to the sudden changes and stress caused by the COVID-19 pandemic.

## Method

### Participants

A total of 383 parents aged 21–50 years participated in this study in Central China. Due to the social distancing policy at the time of the study, all participants were recruited and completed the study online. As this study tended to focus on parents of children aged 3–6, the data were cleaned according to guardianship role and child age. Participants who were not immediate family members of the child (n = 7) and whose child was younger than 3 or older than 6 years (n = 86) were excluded from the data analysis.

The demographic information of the participants and their families is shown in Table 1. The final sample included 290 Chinese parents (256 mothers and 34 fathers) with at least one child aged 3–6. Most parents (68.6%) lived with their partners, with a small proportion (21.4%) living with older people. Almost half of the parents (43.1%) were working at home at the time of data collection and more than half were not working at home (56.9%). The results of the chi-square test showed that the samples of different parental roles did not differ by age (χ^2^ = 4.83, *p* = 0.089) or education (χ^2^ = 5.02, *p* = 0.17).

**Table 1:** Description of participants and their families.

Variables	*N*	%
*Parent*		
Mother	256	88.3
Father	34	11.7
*Age*		
21–30	62	21.4
31–40	199	68.6
41–50	29	10
*Education background*		
High school	63	21.7
Junior college	95	32.8
Bachelor degree	123	42.4
Master's degree	9	3.1
*Work status*		
Working from home	125	43.1
Not working from home	165	56.9
*Employment areas*		
Education	50	17.3
Business	82	28.3
Health	25	8.6
Science & Engineering	11	3.8
Arts	3	1
Law	5	1.7
Homemaker	67	23.1
Graduate student	1	0.3
Administration	22	7.6
Freelancer	6	2.1
Other	18	6.2
*People living at home*		
One or more children < 5 years	82	28.3
One or more children 5–12 years	94	32.4
One or more adolescent/young adult	31	10.7
Other parent/partner	199	68.6
Grandparents	62	21.4
Other	5	1.7

### Procedure

After obtaining the Ethics approval at Xi'an Jiaotong Liverpool University, we collected data online via a Chinese online platform (Wenjuanxing) in, when social distancing measures were still in effect in many Chinese cities. First, we obtained consent forms from the directors and teachers of the preschools, and they supported the research team in sending the research flyers to parents via email. Informed consent was obtained from all the participants before starting the survey. The parents received the survey link via email and were able to voluntarily finish and submit it online. In this process, parents could withdraw at any time according to ethics. All the methods were implemented in accordance with relevant guidelines and regulations.

### Measures

The research tool was developed based on previous studies^27,29^ which examined parents’ perceptions of their own parenting in relation to six interrelated factors—positive parenting, inconsistent discipline, positive relationships, positive emotions, self-efficacy, and routine management—and aimed to learn about parent–child activities in Chinese families with children aged 3–6 following social distancing measures.

#### Demographic information

Participants were asked to provide personal information, including their family role (mother, father, other), age (select a group with a 10-year gap), educational background (high school, junior college, bachelor’s degree, master's degree, or other), occupational field (Education, Business, Health, Science & Engineering, Arts, Law, Homemaker, Graduate student, administration, freelancer, or other), and people living with the family (one or more children under 5, one or more children aged 5–12, one or more adolescents/young adults, other parent or partner, grandparents, or others). All of these questions were single-choice, except for the multiple-choice question on family members living together. Participants who chose the answer ‘other’ were asked to add a specific description and explanation. In addition, they were asked to choose whether this was their first experience of being in lockdown and their working status during that period (working from home, not working from home). If the participants chose ‘working from home’, they were also asked to report how efficiently they worked during this period on a scale of 1–5 (*1* = *not productive at all, 5* = *very productive*).

#### Time spent by parents on childcare

Participants reported the time spent on childcare activities before and during the lockdown, respectively, on a scale of 1–5 *(1* = *100%, 5* = *0%*).

#### Perceptions of parenting

Participants scored the 18 descriptors according to their frequency of occurrence *(5-point scale, 1* = *never, 2* = *seldom, 3* = *sometimes, 4* = *often, 5* = *always*) using the past 48 h as a reference. These items were related to the following six categories: positive parenting, inconsistent discipline, routine management, positive relationships, positive emotions, and self-efficacy. Each category contained three items. The first two categories were designed based on the Alabama Parenting Questionnaire-Short Form developed by Elgar et al.^30^, and the last four categories were expanded from the items provided by Ilari et al.^29^.

The descriptions of positive parenting related to parental approval and praise of the child (e.g., ‘You let your child know that she/he is doing well at something; You praise your child when he/she does well’). Inconsistent discipline corresponded to the parent's agreement and use of punishment with the child (e.g., ‘You threaten to punish your child but do not actually punish him/her; Your child asks you out of punishment after he/she has done something’). The three descriptions of routine management related to the parent's measures to manage the child's daily activities (e.g., ‘You help your child to set his/her daily activity plan; You control the amount of time your child spends in studying’). The three items related to positive emotions assessed the parent's current emotional state (e.g., ‘You feel hopeful about the future; You feel good about your life’). Self-efficacy-related statements reflected parents' confidence in their own parenting behaviours and abilities (e.g. ‘You are confident in taking care of your child's physical health; You are confident in supervising your child's daily activities’).

#### Children's engagement in extracurricular activities

Participants reported the average amount of time their child spent on extracurricular activities each week before and during the lockdown period and selected from a list of online and offline extracurricular activities in which their child had participated. If the option ‘other’ was selected, parents were asked to add specific activity names.

#### Parent–child activities

Participants reported on the types of activities they participated in with their children during the lockdown and selected their three favourite activities to participate in with their children. If the parent–child activities that they have participated in have not been listed, parents could choose ‘other’ and provide specific names.

#### Reflections on parent–child relationships and parenting experiences

Participants were invited to answer the following two questions.

1) In what ways do you believe that the COVID-19 pandemic has changed your relationship with your child/children?

2) Would you like to share your experience of parenting during the lockdown period?

### Data analysis

All statistical analyses were performed using SPSS (26.0). Cronbach's alpha was used to analyse the reliability of the six parenting components (Table 2). One-way MANOVAs were used to analyse the relationships between parental role, age, educational background, perceived work productivity, and the six components of parenting. Chi-square tests were used to examine the relationships between parental roles in terms of age and educational background. Paired-sample t-tests were used to compare the time that children spent on extracurricular activities before and during the lockdown period. Wilcoxon signed-rank tests were used to analyse the differences in parental time devoted to childcare before and during the lockdown period. In addition, content analysis, which involves the subjective interpretation of textual content^33^^31^, was used to analyse two open-ended questions at the end of the questionnaire. This method facilitates the transformation of unstructured textual content, which is a jumble of parental responses, into structured data, effectively inferring the meaning of the content and drawing conclusions^34^^32^. Parents' responses were categorised based on implied affective tones (positive, negative, and neutral) and topics.

**Table 2:** Descriptive data.

	Mean	SD	*Cronbach's alpha*
Work productivity at home	3.03	0.80	N/A
Positive parenting	4.38	0.61	0.76 (3 items)
Inconsistent discipline	2.76	0.76	0.54 (3 items)
Routine management	3.53	0.71	0.57 (3 items)
Positive relationships	4.14	0.61	0.68 (3 items)
Positive emotions	3.75	0.67	0.62 (3 items)
Self-efficacy	3.65	0.75	0.74 (3 items)

## Results

### Influence of parental role, age, education background and work productivity on parenting

First, we performed a one-way multivariate analysis of variance (MANOVA) to investigate the effects of parents' family roles, age, and educational background on six factors related to parenting (i.e., positive parenting, inconsistent discipline, positive relationships, positive emotions, self-efficacy, and routine management) during the lockdown period. Results indicated that parents of different age ranges and levels of educational attainment did not show any differences in the six parenting categories. However, family role did make a difference in terms of parenting behaviours, with significant differences shown in self-efficacy, *F* (1, 288) = 5.53, *p* < 0.05, partial η^2^ = 0.02. Specifically, fathers (*M* = 3.93) had a much higher level of parenting self-efficacy than mothers (*M* = 3.61) during the pandemic.

The parents reported moderate levels of productivity at home (*M* = 3.03, SD = 0.803). The results of the one-way MANOVAs examining the variability between parents' work productivity and their parenting indicate significant variability (*p* < 0.05) between positive emotions, routine management, and parental work productivity. Specifically, perceived work productivity for positive parenting (*F* (4, 285) = 2.89, *p* = 0.02) and routine management (*F* (4, 285) = 2.83, *p* = 0.03) both showed a 0.05 level of significance. However, parents’ work productivity at home was not significant (*p* > 0.05) for inconsistent discipline, positive parenting, positive relationships, or self-efficacy.

We also examined whether family members living together during the pandemic affected their parenting practices. Interestingly, the results of the one-way MANOVA revealed that in families with grandparents, parents showed lower levels of positive relationships (*F* (1, 288) = 6.49, *p* = 0.01) and self-efficacy (*F* (1, 288) = 7.62, *p* = 0.01) but not in the other four dimensions.

### Changes in children's participation in extracurricular activities

Another question of interest was the children’s participation in extracurricular activities. Before social distancing was mandated, children spent an average of 7.98 h per week (SD = 8.50) engaging in extracurricular activities. With the shutdown of schools and services, the number of hours that children spent on extracurricular activities showed a slight decrease to 7.87 h (SD = 9.97). A paired-sample t-test revealed that this difference was not statistically significant (*t* (288) = 0.22, *p* = 0.829. In other words, in our current sample, children generally spent a similar amount of time engaging in extracurricular activities before and after social distancing measures were implemented for the pandemic.

**Figure 1:**
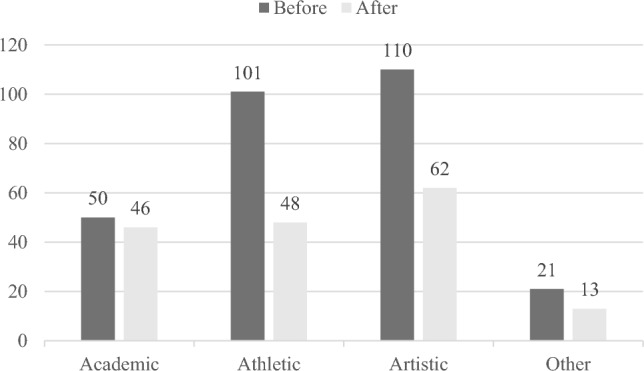
Children's engagement in extracurricular activities before and after COVID-19 quarantine.

In addition to analysing the amount of time children spent on extracurricular activities, the current study also analysed changes in the types of such activities before and during the lockdown. Extracurricular activities were divided into four categories: ‘academic’, ‘athletic’, ‘artistic’, and ‘others’ (Fig. 1). Athletic activities decreased the most (approximately 52%), artistic and other extracurricular activities decreased by 44% and 38%, respectively, while academic activities showed the least decrease at only 8%. Within each category, children's participation in all academic activities increased as follows: language tuition (27%), mathematics tuition (43%), English tuition (80%), reading training (45%), and programming classes (20%). Within the athletic category, all activities showed a decreasing trend, except for badminton (1 activity reported at both time points), running (from 2 to 3), and dance (from 5 to 11), showing an increase. The most popular activity, basketball, decreased by approximately 48%, from 52 to 27. A decrease in all kinds of artistic activities was observed after the lockdown, and the most popular activity, drawing, decreased by almost 40% from 78 to 47. Among the other extracurricular activities in which the children participated, baking, cooking, jumping ropes, and other leisure activities decreased.

### Parent–child activities during the COVID-19 pandemic

Unsurprisingly, parents spent more time on childcare and nurturing at home during the pandemic. The results of the Wilcoxon signed-rank test suggested that parents spent more time on childcare during the pandemic than before the social distancing measures were implemented, *z* = − 6.63, *p* < 0.001.

**Figure 2:**
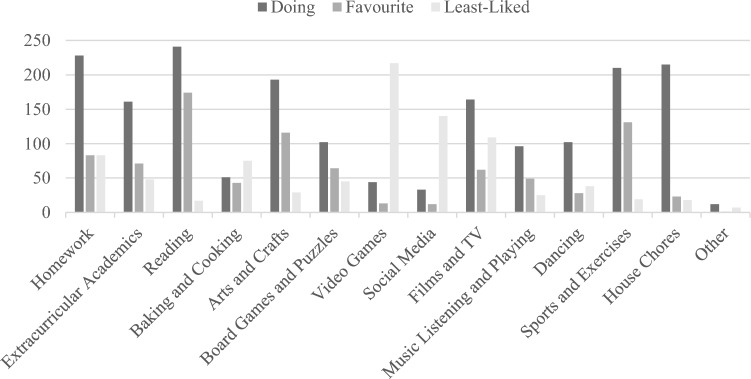
Parent–child activities during COVID-19 quarantine.

Approximately 90% of the parents reported that they spent more time and were engaged in more activities of various types with their children at home during the pandemic. Figure 2 presents the frequency of engaging in such activities. Reading (*N* = 241) appeared to be the most frequent activity of parents and children at home, followed by homework (*N* = 228), house chores (*N* = 215), and sports and exercise (*N* = 210). Parents also enjoyed reading with children the most, followed by doing sports and exercise (*N* = 131), and arts and crafts (*N* = 116). Playing video games, using social media, and watching films and TV, however, featured parents’ least liked activities to do with their children. At home, parents reported that they had to support children with their homework and finish reading with them. Moreover, parents did not like their children spending much time on electronic devices when staying at home.

At the end of the questionnaire, parents shared thoughts about their parent–child relationship and parenting experiences during the lockdown period if they wished to do so. As a result of the content analysis shown in Table 3, the answers to the collected open-ended questions contained 167 items grouped into 3 categories based on relevant themes and implied emotions: positive (*N* = 105), negative (*N* = 33), and neutral (*N* = 29) elements. Among the positive themes extracted, empathetic views toward parents included having more parent–child time (23.4%), understanding each other better (15.6%), and having a stronger parent–child relationship (15%). Regarding negative themes, parents most frequently reported being exhausted and impatient (4.2%), followed by conflict with their children during the pandemic (3.6%).

**Table 3:** Themes extracted from open-ended questions.

Theme	Count	Percentage	Quotes
*Positive elements*			
Spending more time together	39	23.4	*We have more time to play games and do sports together*
Learning more about each other	26	15.6	*I can know more about my child's study, life and growth*
Improving parent–child relationship	25	15	*We have a closer relationship*
Child become more independent and positive	6	3.6	*My child keep positive during this period*
Parents become more patient	4	2.4	*I become less anxious and more relaxed about parenting*
Getting more free time	2	1.2	*We have more free time, my child and I don't have to study and work at fixed hours*
Child become more active in study	2	1.2	*Child learns more actively and seriously*
Child learning valuable life lessons	1	0.6	*Letting child help clean up the house can cultivate his sense of family responsibility*
Total	105	62.9	
*Negative elements*			
Stress, exhaustion, impatience	7	4.2	*Parents who spend too much time with their child are prone to depression*
Conflicts between child and parents	6	3.6	*I don't think I can get along well with my boy for more than three hours, especially when it comes to homework*
Tough parenting tasks	4	2.4	*Supervising child's homework gives me a headache*
Decline in recreation and outdoor activities	4	2.4	*We can't go outside*
Balancing parenting, work and own life	3	1.8	*I don't have time to do my own things!*
Multiple challenges at once	3	1.8	*I not only took care of child's daily life, but also took on the role of teacher*
Chaotic Time planning	3	1.8	*I don't know how to organize my child's daily schedule*
Noisy, clingy kids	2	1.2	*The child makes too much noise at home every day*
Concerns with health	1	0.6	*It is difficult to see the doctor and my child had a cough for several days*
Total	33	19.8	
*Neutral elements*			
No change	4	2.4	*Because we spend so much time together, I don't realize any change*
Words of advice	25	15	*Just let the child be happy in the early childhood*
Total	29	17.4	

## Discussion

In this study, we explored the parenting cognition and practices of Chinese parents of children aged 3–6 during the COVID-19 lockdown period. Based on the results of the data analysis, five key findings are summarised. First, mothers demonstrated significantly lower levels of self-efficacy than fathers. According to the ecological systems theory, proximal and distal systems interact with each other and jointly affect the outcomes of members of the system^23^. Consequently, parental self-efficacy at work may be generalised and transferred to the perceived efficacy of managing relationships with children at home. Research has suggested that women tend to report higher levels of anticipated work-family conflict, yet lower efficacy in managing these conflicts than men (e.g.,^33^. Therefore, mothers might have perceived lower efficacy at work due to the interruption of the COVID-19 pandemic and more stress in balancing their work and family life, which led to the lower self-efficacy of parenting reported by mothers compared to fathers.

Another possible reason for the differential self-efficacy reported by mothers and fathers is the cultural background of this study. Xiao^34^ has found that Chinese mothers show lower self-efficacy in parenting tasks because of, for example, their relationship with grandparents. Research has demonstrated that a harmonious relationship between parents and grandparents contributes to parental efficacy (Li et al 2019)^35^. Among the common patterns of parents living with grandparents in China, the majority live with their parents, who tend to have a more harmonious relationship with their father and, thus, may better contribute to the father's feeling of being a good parent. Therefore, to increase mothers' perceptions of parenting efficacy, emotional support from family members must be increased. Fathers and mothers, however, did not differ on other aspects of parenting during the pandemic, which may suggest that the ensuing stress had similar effects on them. In addition, our results suggest that parents, in general, show decreased levels of positive relationships and self-efficacy in families with grandparents, which might have emphasised the conflict between different generations living together during the COVID-19 pandemic. Previous studies have suggested that high-intensity childcare may worsen the health conditions of co-residing grandparents, such that grandparents living with parents and grandchildren in three-generation families may experience rapid health decline in China^36^. Given the highly stressful circumstances under quarantine measures, three-generation families may have more conflict regarding childcare during this special period, leading to less positive relationships between parents and children, as well as lower self-efficacy in parenting.

Second, highly productive parents had higher levels of positive emotions and routine management. In the context of irregular working hours, high productivity means that parents can transfer the time and energy saved at work to their parenting practices. This might be because parents who are more productive at work are more likely to actively engage in parent–child interaction and life management, which, in turn, leads to a better parent–child relationship. The relationship between productivity and daily management can be explained by one parent’s answer at the end of the questionnaire: ‘During the lockdown, I make my own work and study plan every day and I also help my child to make his study plan. We are both more responsible for our duties’. This suggests that parents who are more productive at work may also tend to have a regular work schedule, and work habits are exemplified for their children in parent–child interactions. Therefore, parents with higher work productivity may be better at managing their children’s routines at home. Organised and productive family life may benefit parental psychological adjustment and increase positive emotions during a pandemic. Furthermore, this study did not find a significant effect of work productivity on positive parenting, positive relationships, self-efficacy, or inconsistent discipline. Given the positive effects of higher productivity on family functioning, more social support should be provided to parents at the company and community levels to enhance their work at home.

Third, we also found that most sports and arts activities that children participated in during the lockdown period were significantly reduced. This was because the stay-at-home policy resulted in children not having access to many necessary spaces, equipment, or other resources for extracurricular activities. In a review study, Stockwell et al. ^37^ reported that both adults and children across different countries reported decreased physical activity and increased sedentary behaviours during the COVID-19 lockdown compared to the pre-pandemic period. However, there was an increase in children participating in running and dancing activities, which may be because these two activities do not require much in the way of space and equipment. Physical activity can benefit both physical and mental health, and maintaining physical activity was found to buffer adolescents’ depressive symptoms during the COVID-19 pandemic ^38^. As many parents realise that physical activity can be effective in improving their children's physical and mental health in a stressful environment, they may advise their children to become more involved in running and dancing despite the constraints. A study in France and Switzerland also found that people reported more walking and moderate physical activities yet less rigorous activities during the lockdown period ^39^. In the face of such challenges, schools, teachers, and arts training providers could develop more technologies to provide remote instructions on extracurricular activities, such as mobile phone software that supports teachers in connecting remotely to mirror instruction on movement standards ^40^. Parents could also use existing resources at home to provide alternatives for their children,for example, one parent said: ‘To keep my child practising drumming, I placed some pillows on the sofa in the standard position and had him simulate drumming by hitting the pillows’.

Fourth, despite limited extracurricular activities, children's overall participation length did not decrease significantly, which differs from the findings of Ilari et al.^29^. One possible explanation is that, although a large proportion of extracurricular activities were not available due to restrictive conditions, such as inadequate space and equipment resources, children extended their participation in other indoor extracurricular activities to compensate for this lack of outdoor experience. The main type of extracurricular activities that children participated in during the lockdown period was likely academic training, as parents reported more examples of children participating in language, mathematics, English, reading, and programming training sessions. Almost three years have passed since the outbreak of COVID-19. Subsequently, various online platforms that assist children in learning at home have made significant strides, with subject-related distance tutoring being updated. This means that children have more support, as well as more options when engaging in various online academic training courses at home.

Fifth, due to the social distancing policy, parents spent more time with their children at home. Several provinces and municipalities issued stricter precautionary measures to prohibit people from leaving their buildings. In this restrictive environment, parents and children spend almost the entire day at home, inevitably having more time together and more opportunities for interaction. This was also confirmed in a study by Huang and Tsai ^41^, in which the majority of parents reported spending more time with their children during COVID-19. Parents and children also have more opportunities to participate in parent–child activities. Similar to children's participation in extracurricular activities, parent–child activities during the lockdown period were characterised by low requirements for space and equipment. Parents preferred reading, sports and exercise, and arts and crafts. The reason for choosing reading activities may be Chinese parents' pursuit of developing their children's excellent study habits and improving their academic performance. Their academic expectations regarding their children may be high, yet the latter may also benefit from supportive home-based parental involvement in academic studies ^42,43^. As indicated by our participants, ‘(The lockdown experience) facilitated our academic communication’. In addition, the popularity of sports may be the result of parents' conscious desire to boost their own and their children's physical and mental health. The support for arts and crafts may be due to parents' awareness that such activities, while fostering aesthetics and creativity, also help to improve children's resilience and adaptability during periods of high pressure ^44^.

Parents were concerned about the use of electronic devices. The top three parent–child activities selected by parents as their least favourite were all related to electronic devices: video games, social media, movies and television. This attitude was also found in the open questions, where some parents reported that their child's use of electronic devices had increased significantly, giving rise to a range of other problems such as irregular sleep time, addiction to online games, deteriorating eyesight, and parent–child conflicts. The extant literature also suggested that adolescents tended to use more social media and play more games during the COVID-19 pandemic, which may negatively affect their healthy behaviours, such as sleep and exercise ^45^. Several parents, however, used positive parenting measures to address this challenge: ‘I find that he is now addicted to playing video games. I deliberately invited him to exercise and perform chores with me to reduce his access to the electronics,’ and ‘I also play video games with him to monitor his behaviour’. Active parental involvement has been shown to reduce the negative effects of excessive screen exposure on children's physical and mental health (Meoded ^46^. In addition, one parent stated, ‘The child has to use his mobile phone or tablet computer for online classes, and there is no way to solve this problem by controlling the usage of devices’. Follow-up research should focus on effective prevention and interventions to manage children's use of electronic devices in specific contexts.

The results of this study confirm the theories explained in the previous section. The impact of a lockdown on the family system and family members during a pandemic is complex and results from a combination of distal and proximal factors. The stressors spawned by restrictive policies are constantly transmitted and changed within the family system and affect the mental health of parents and children. The current study found that parental work productivity was associated with positive emotions and management of child routines at home during the COVID-19 pandemic, suggesting that parental adjustment during the lockdown period may perpetuate the family system and affect parent–child interactions. Our results also indicated that parents' perceptions of parenting and parenting behaviours have partly changed in this context. They spend more time with their children at home and feel exhausted and stressed when balancing their work and family lives, suggesting that more social and policy support is needed to help families recover after the COVID-19 pandemic. The proposed policy must involve the combined efforts of several groups, including families, children, schools, institutions, and enterprises. Attention and support from society also contribute to the well-being of families, including financial assistance, family care services, family education guidance, distance learning training, and family conflict mediation services.

### Limitations and future research

Despite the interesting results of this study, it has some limitations. First, the number of female participants (mothers) was much larger than that of male participants (fathers). Although mother respondents are usually found in the research field (Ilari et al., 2022), future research could consider recruiting more male respondents because their perceptions and parenting skills might be different. Second, we only partially collected demographic information on the participants; future studies could include their socioeconomic status, ethnicity, race, and so on. Several studies have already demonstrated that social class plays an important role in parenting (e.g. Stein & Breckenridge, 2021); subsequent studies could also include this factor to examine whether parents from different social classes demonstrate different parenting practices in the Chinese sample. Ethnic groups may also have a significant effect on parenting behaviours and perceptions owing to China's ethnic diversity, and future studies could test this hypothesis. Third, the pandemic lockdown policies varied over time as the pandemic progressed. As China's policy changes, parents’ perceptions could differ from the increased physical and mental family support services available to families at the micro-level, which continue to expand (Bronfenbrenner & Morris, 2006), and the issues addressed in this study are soon to become time-sensitive. Future research could focus on parents’ attitudes, extracurricular activities for children, and parent-child activities in the post-epidemic era. Using longitudinal studies, researchers can gain a deeper and more comprehensive understanding of the short- and long-term effects of the epidemic on parents, children, and families.
